# Supplementary material for: Real-Time Pharmacovigilance: Transforming Population-Based Monitoring of Post-Approval Vaccine Safety Through Rapid Cycle Analysis (RCA)—A Review of the Published Literature
Source: Pharmaceuticals (Basel). 2025 Jan 10;18(1):80. doi: 10.3390/ph18010080 (PMC11769534; doi:10.3390/ph18010080)
Supplement: Supplementary file 1 [file pharmaceuticals-18-00080-s001.zip › Table S1.pdf]

Table S1: Key Study Characteristics of Vaccine RCA Studies Conducted for Ascertainment of Safety of COVID-19 Vaccine

Author, Year and Country	Name of Data Source (Type)	Safety Monitoring	Study Period	Study Population	Safety Outcomes	At-risk period	Type of Comparator	Statistical Analysis Method	Frequency of Analysis	Signaling Detection Threshold	Confounding Control Method	Confounding Variables
Goddard, 2023, US	VSD (EHR)	Signal Detection	6/18/2022-3/18/2023	Pediatric, general population	23 pre-specified safety outcomes. 11 Outcomes underwent sequential testing: Appendicitis; Bell's Palsy; Encephalitis, myelitis, or encephalomyelitis; Gullain-Barre syndrome; Immune thrombocytopenia; Kawasaki disease; Pulmonary embolism; Seizures; Stroke, hemorrhagic; Transverse myelitis; Venous thromboembolism; 12 outcomes not included in sequential testing: Acute disseminated encephalomyelitis; Acute myocardial infarction; Cerebral venous sinus thrombosis; Disseminated intravascular coagulation; Myocarditis/Pericarditis; Stroke, ischemic; Thrombosis with thrombocytopenia syndrome;	1-21 days	Vaccinated concurrent comparator	RCA with poisson regression sequential testing	Weekly	Yes	stratification by confounding variables	VSD site, age, sex race, ethnicity, and calendar day

					Thrombotic thrombocytopenic purpura;<br>Acute respiratory distress syndrome;<br>Anaphylaxis;<br>MIS-C;<br>Narcolepsy or cataplexy							
Lloyd, 2022, US	Optum, HealthCore, CVS Health (Claims)	Signal Detection	12/11/2020-01/22/2022	Pediatric and Adult, general population	Acute myocardial infarction, Deep vein thrombosis, pulmonary embolism, disseminated intravascular coagulation, non-hemorrhagic stroke, hemorrhagic stroke, immune thrombocytopenia, myocarditis/pericarditis, GBS, Bell's palsy, enchephalomyelitis/encephalitis, anaphylaxis, transverse myelitis, narcolepsy, appendicitis, common thromboses with thrombocytopenia, unusual site thromboses with thrombocytopenia	1-42 Days	Historical (background) rate: general population or influenza vaccinated population	Poisson Maximized Sequential Probability Ratio Test	Biweekly or Monthly	Yes	Stratification by age and sex	Age and sex
Wong, 2023, US	US Centers for Medicare and Medicaid Services (CMS) (Claims)	Signal Detection	12/11/2020-1/15/2022	Elderly, general population	Acute myocardial infarction, Deep vein thrombosis, pulmonary embolism, disseminated intravascular coagulation, non-hemorrhagic stroke, hemorrhagic stroke, immune thrombocytopenia,	1-28 days;<br>1-42 Days	Historical (background) rate: general population	Poisson Maximized Sequential Probability Ratio Test (PMaxSPRT); secular trends adjustment	Weekly	Yes	stratification of standardized variables (Nursing home, age, sex, race)	delay in claims processing and standardized by nursing home residency status,

					myocarditis/pericarditis, GBS, Bell's palsy, enchephalomyelitis/encephalitis, tranverse myelitis, narcolepsy, appendicitis							age, sex, and race
Luo, 2023, US	Veterans Health Administration (Claims)	Signal Detection	12/14/2020-10/9/2022	Adult, general population	Myocarditis and Pericarditis	1-21 Days	Historical vaccine recipient controls	Poisson-based maximized sequential probability ratio testing to adjust for sequential testing; O/E analysis	Weekly	Not specified	None Specified	None
Hause, 2022, US	VSD (EHR)	Signal Detection	10/31/2021-2/26/2022	Pediatric, general population	Acute respiratory distress syndrome, anaphylaxis, MISC, narcolepsy, myocarditis and pericarditis, acute disseminated encephalomyelitis, acute myocardial infarction, appendicitis, Bell's palsy, cerebral venous sinus thrombosis, disseminated intravascular coagulation, encephalitis, myelitis, or encephalomyelitis, Guillain-Barre syndrome, immune thrombocytopenia Kawasaki disease, pulmonary embolism, seizure, hemorrhagic stroke, ischemic stroke,	1-21 Days	Vaccinated concurrent comparator	Poisson regression sequential testing	Weekly	Yes	None Specified	age, sex, calendar day, site, and race and ethnicity groups

					thrombosis with thrombocytopenia syndrome, thrombotic thrombocytopenic purpura, transverse myelitis, venous thromboembolism							
Klein, 2021, US	VSD (EHR)	Signal Detection	12/14/2020-6/26/2021	Pediatric and Adult, general population	acute disseminated encephalomyelitis, anaphylaxis, encephalitis/myelitis, Guillain-Barré syndrome, immune thrombocytopenia, Kawasaki disease, narcolepsy, seizures, transverse myelitis, appendicitis, Bell palsy, acute myocardial infarction, acute respiratory distress syndrome, disseminated intravascular coagulation, multisystem inflammatory syndrome in children and adults, myocarditis/pericarditis, pulmonary embolism, stroke [hemorrhagic and ischemic], thrombotic thrombocytopenic purpura, venous thromboembolism, cerebral venous sinus thrombosis, thrombosis with thrombocytopenia syndrome, and a younger subgroup of	1-21 Days	Vaccinated concurrent comparator Unvaccinated concurrent comparator	Poisson regression sequential testing	Weekly	Yes	Stratification by 5-year age group, sex, race and ethnicity groups, site, and calendar day.	5-year age group, sex, race and ethnicity groups, site, and calendar day.

					the myocarditis/pericarditis outcome							
Hu, 2023, US	Optum, HealthCore, CVS Health; (Optum and CVS Health were supplemented with data from local and state immunization information systems (Claims)	Signal Detection	EUA – 6/25/2022 for Optum, 5/6/2022 for HealthCore, 5/31/2022 for CVS Health	Pediatric, general population	Acute myocardial infarction, Anaphylaxis, appendicitis, Bell's Palsy, common site thrombosis with thrombocytopenia, DVT, DIC, encephalitis or encephalomyelitis, febrile seizures, GBS, hemorrhagic stroke, immune thrombocytopenia, Kawasaki disease, MIS in peds, Myocarditis, Pericarditis, narcolepsy, non-hemorrhagic stroke, PE, seizures, transverse myelitis, unusual site thrombosis with thrombocytopenia	1-42 Days; 1-28 days; 1-21 days; 1-7 days, 0-7 days, 0-1 days	Historical (background) rate: general population	Poisson maximized sequential probability ratio test	Monthly	Yes	Adjusted historical rates by age and sex	Age and sex
