# Supplementary material for: Real-Time Pharmacovigilance: Transforming Population-Based Monitoring of Post-Approval Vaccine Safety Through Rapid Cycle Analysis (RCA)—A Review of the Published Literature
Source: Pharmaceuticals (Basel). 2025 Jan 10;18(1):80. doi: 10.3390/ph18010080 (PMC11769534; doi:10.3390/ph18010080)
Supplement: Supplementary file 1 [file pharmaceuticals-18-00080-s001.zip › Table S2.pdf]

Table S2: Key Study Characteristics of Vaccine RCA Studies Conducted for Ascertainment of Safety of Non-COVID-19 Vaccines

Author, Year and Country	Name of Data Source (Type)	Safety Monitoring	Vaccine Studied	Study Period	Study Population	Safety Outcomes	At-risk period	Type of Comparator	Statistical Analysis Method	Frequency of Analysis	Signaling Detection Threshold	Confounding Control Method	Confounding Variables
Sundaram, 2022, US	VSD (EHR)	Signal Detection	Human papilloma virus (HPV)	01/01/2007-01/02/2021	Pediatric and Adult, general population	Guillain-Barré syndrome (GBS), chronic inflammatory demyelinating polyneuropathy (CIDP), stroke	1-42 days for GBS; 1-180 days for CIDP; 0-42 days for stroke	Historical (background) rate: other vaccinated	1-sided Fisher's exact test and exact logistic regression analysis	Not specified	Yes	Stratification	Age and Sex
Arya, 2019, US	Medicare (Claims)	Signal detection and signal evaluation	Influenza	2015-2016 and 2016-2017 flu seasons (exact dates not specified)	Older adult, general population	GBS	1-42 days; 8-21 days (attributed case self-controlled risk interval [SCRI] analysis); 43-84 days (post vaccination control window)	Historical (background) rate: general population	Updating Sequential Probability Ratio Test, conditional logistic regression	Not specified	Not specified	RCA stratification by age, sex, race, region, standard and high dose flu vaccines, and concomitant vaccines	Age, sex, race, region, standard and high dose flu vaccines, and concomitant vaccines
Donahue, 2019, US	VSD (EHR)	Signal detection	HPV	10/04/2015-10/07/2017	Pediatric and Adult, general population	Anaphylaxis, allergic reaction, appendicitis, GBS, chronic inflammatory demyelinating polyneuropathy, injection site reaction, pancreatitis, seizure, stroke, syncope, and venous thromboembolism	1-42 days for most outcomes, a few outcomes were shorter or longer	Historical (background) rate: general population or influenza vaccinated population Concurrent comparator (other vaccines); SCRI	Poisson-based MaxSPRT, conditional maximized sequential probability ratio test (CMaxSPRT), and exact sequential analysis (ESA)	Weekly	Yes	stratification of age, sex, site, and week of vaccine administration, and exposed vs unexposed	Age, sex, site, and week of vaccine administration, and exposed vs unexposed
Perez-Vilar, 2021, US	VSD (EHR)	Signal detection and signal evaluation	Influenza	07/01/2018-4/3/2019	Any, general population	GBS	1-42 days; 8-21 days	Historical (background) rate: influenza vaccinated SCRI	Poisson-based maximized sequential probability ratio test (maxSPRT) AND sequential method binomial maxSPRT	Weekly	Yes	Not Specified	Not Specified
Sun, 2021, US	Regional Health Information Platform (RHIP) (EHR)	Signal detection and signal evaluation	Enterovirus 71 (EV71)	1/1/2016-12/31/2019	Pediatric, general population	Febrile seizures	0-7 days	SCRI; Historical (background) rate: general population	MaxSPRT(binomial-based version (BMaxSPRT) for the SCRI design; the Poisson-based (PMaxSPRT); or Poisson-based conditional version	Weekly	Yes	None Specified	Age and season

									(CMaxSPRT) for the current vs. historical cohort design				
