# Supplementary material for: Real-Time Pharmacovigilance: Transforming Population-Based Monitoring of Post-Approval Vaccine Safety Through Rapid Cycle Analysis (RCA)—A Review of the Published Literature
Source: Pharmaceuticals (Basel). 2025 Jan 10;18(1):80. doi: 10.3390/ph18010080 (PMC11769534; doi:10.3390/ph18010080)
Supplement: Supplementary file 1 [file pharmaceuticals-18-00080-s001.zip › Table S3.pdf]

Table S3: Search String and Strategy for Literature Identification

A comprehensive and systematic search was conducted using a combination of controlled vocabulary, text words and keywords for the past 5 years in Embase and Medline via Ovid. The search consists of the following concepts: vaccines, safety surveillance/adverse event reporting, and rapid cycle analysis. Text words for sensitive concepts like safety were limited to title and abstract while combined logically with extensive indexing and headings. Similarly, text words for vaccines were limited to title and abstract to maintain the specificity of the subheadings attached to vaccine related indexing. However, the concept of rapid cycle analysis was extended beyond title and abstract to capture potential headings and author keywords, as no indexing currently exists for this concept. The combination of these facets balances sensitivity and specificity.

The search was performed in OvidSP on August 8, 2024. Results were limited to the time period January 1, 2018 to July 31, 2024, and were limited to English using the Ovid language filter. A separate facet for animal studies was extrapolated from the CADTH validated filter as an exclusion to limit the results to human data as much as possible. Results were deduplicated between Medline and Embase using the Ovid deduplication prompt.

Databases

Literature Database Name	Years Covered	Date Searched	Date Limits Applied
Embase	1974 to Current	08Aug2024	01Jan2018-31Jul2024
Ovid Medline	1946 to Current	08Aug2024	01Jan2018-31Jul2024

Search Strategy

Database: Ovid MEDLINE(R) ALL <1946 to August 07, 2024>, Embase <1974 to 2024 August 07>
Search Strategy:
1 exp vaccine/ae, an, to, pv or exp vaccination/ae use oemezd [Adverse Drug Reaction, Drug Analysis, Drug Toxicity, Special Situation for Pharmacovigilance] (92457)
2 exp Vaccines/ae, an, po, to or exp Vaccination/ae, co use medall [Adverse Effects, Analysis, Poisoning, Toxicity] (88206)
3 (vaccine* or vaccination* or immuniz* or immunis*).ti,ab. (1091625)
4 or/1-3 [vaccine] (1107766)
5 exp drug safety/ or safety/ or exp patient safety/ or product safety/ or product vigilance/ or exp adverse drug reaction/ or adverse event/ or drug surveillance program/ or postmarketing surveillance/ or exp drug monitoring/ or signal detection/ or active surveillance/ or exp

pharmacovigilance/ use oemezd (1976222)
6 Pharmacovigilance/ or Drug Monitoring/ or Safety/ or Patient Harm/ or Patient Safety/ or Product Surveillance, Postmarketing/ or exp "Drug-Related Side Effects and Adverse Reactions"/ or exp Population Surveillance/ use medall (1454852)
7 Long Term Adverse Effects/ or exp Drug Interactions/ or unspecified side effect/ or Drug Induced Disease/ or exp Application Site Reaction/ or exp Drug Eruption/ or exp Drug Eruptions/ or exp Drug Hypersensitivity/ or exp Side Effect/ or exp Postmarketing Surveillance/ or drug surveillance program/ or drug fatality/ or chemically-induced disorders/ (1511973)
8 (safety or surveillance or monitor* or signal* or surveilling).ti,ab. (8209405)
9 exp adverse event/ or (Adverse* or ADE or ADEs or ADR or ADRs or SAE or SAEs or TRAE or TRAEs or TEAE or TEAEs or irAE or irAES or AESI or ((serious or nonserious or non serious or severe or treatment or emergent) adj2 (ae or aes))).ti,ab. (2782829)
10 ((safety adj (risk* or assessment*)) or (risk adj2 assessment*)).ti,ab. (281522)
11 (pharmacovigilan* or pharmaco vigilan*).ti,ab. (22441)
12 (drug adj3 (safety or surveillance or monitor* or surveilling)).ti,ab. (86180)
13 (signal adj3 (detect* or percept* or monitor* or surveillance or surveilling)).ti,ab. (48028)
14 ((side or undesirable or unwanted or adverse or serious or nonserious or non serious) adj2 (effect* or reaction* or event* or outcome*)).ti,ab. (2347263)
15 or/5-14 [safety] (11994069)
16 (rapid cycle analysis or rapid cycle evaluation or rapid signal identification*).tw,hw,kw,kf. (60)
17 (rapid adj3 (monitor* or surveill* or analys* or analyz* or analytic*)).tw,kw,kf. (37463)
18 (sequential* adj3 (monitor* or test* or analys* or analyz* or method* or surveill*)).tw,kw,kf. (47347)
19 (RCA or VSD).tw,kw,kf. (40946)
20 ((real time or near real time) not ("real time PCR" or "RT PCR" or real time Polymerase Chain Reaction)).tw,kw,kf. (449122)
21 vaccine safety datalink.tw,kw,kf. (570)
22 or/16-21 [RCA] (571989)
23 4 and 15 and 22 (3653)
24 exp animals/ (59632008)
25 exp animal experimentation/ or exp animal experiment/ (3239329)
26 exp models animal/ (2533572)
27 nonhuman/ (7829820)
28 exp vertebrate/ or exp vertebrates/ (58061505)
29 (rat or rats or mouse or mice or swine or porcine or murine or sheep or lambs or pigs or piglets or rabbit or rabbits or cat or cats or dog or dogs or cattle or bovine or monkey or monkeys or trout or marmoset$1 or equine or chicken* or hamster or poultry or racoon* or duck).ti. and animal experiment/ (1290142)
30 or/25-29 (60582017)
31 exp humans/ (49085833)
32 exp human experimentation/ or exp human experiment/ (681755)
33 or/31-32 (49089335)
34 30 not 33 (11494909)
35 23 not 34 [CADTH humans hedge] (3121)

36 limit 35 to dt=20180101-20240731 use medall [Limit not valid in Embase; records were retained] (828)
37 limit 35 to ep=20180101-20240731 use medall [Limit not valid in Embase; records were retained] (698)
38 limit 35 to dc=20180101-20240731 use oemezd (969)
39 or/36-38 (1800)
40 limit 39 to english language (1766)
41 remove duplicates from 40 (1128)
